# Antiproliferative Activity of Fucan Nanogel

**DOI:** 10.3390/md10092002

**Published:** 2012-09-17

**Authors:** Nednaldo Dantas-Santos, Jailma Almeida-Lima, Arthur Anthunes Jacome Vidal, Dayanne Lopes Gomes, Ruth Medeiros Oliveira, Silvia Santos Pedrosa, Paula Pereira, Francisco Miguel Gama, Hugo Alexandre Oliveira Rocha

**Affiliations:** 1 Laboratory of Biotechnology of Natural Polymers (BIOPOL), Departament of Biochemistry, Federal University of Rio Grande do Norte (UFRN), Natal-RN 59078-970, Brazil; Email: nednaldod@hotmail.com (N.D.-S.); biolottus23@yahoo.com.br (J.A.-L.); arthur_bio@hotmail.com (A.A.J.V.); dayanne_gomes@hotmail.com (D.L.G.); rmo_85@hotmail.com (R.M.O.); 2 Graduate Program in Health Sciences, Federal University of Rio Grande do Norte (UFRN), Natal-RN 59078-970, Brazil; 3 IBB—Institute for Biotechnology and Bioengineering, Centre for Biological Engineering, Minho University, Braga 4704-553, Portugal; Email: silviasantospedrosa@deb.uminho.pt (S.S.P.); paulapereira@deb.uminho.pt (P.P.); fmgama@deb.uminho.pt (F.M.G.)

**Keywords:** nanogels, cancer cells, sulfated polysaccharides, *Spatoglossum schröederi*

## Abstract

Sulfated fucans comprise families of polydisperse natural polysaccharides based on sulfated L-fucose. Our aim was to investigate whether fucan nanogel induces cell-specific responses. To that end, a non toxic fucan extracted from *Spatoglossum schröederi* was chemically modified by grafting hexadecylamine to the polymer hydrophilic backbone. The resulting modified material (SNFuc) formed nanosized particles. The degree of substitution with hydrophobic chains was close to 100%, as estimated by elemental analysis. SNFfuc in aqueous media had a mean diameter of 123 nm and zeta potential of −38.3 ± 0.74 mV, as measured by dynamic light scattering. Nanoparticles conserved their size for up to 70 days. SNFuc cytotoxicity was determined using the MTT assay after culturing different cell lines for 24 h. Tumor-cell (HepG2, 786, H-S5) proliferation was inhibited by 2.0%–43.7% at nanogel concentrations of 0.05–0.5 mg/mL and rabbit aorta endothelial cells (RAEC) non-tumor cell line proliferation displayed inhibition of 8.0%–22.0%. On the other hand, nanogel improved Chinese hamster ovary (CHO) and monocyte macrophage cell (RAW) non-tumor cell line proliferation in the same concentration range. The antiproliferative effect against tumor cells was also confirmed using the BrdU test. Flow cytometric analysis revealed that the fucan nanogel inhibited 786 cell proliferation through caspase and caspase-independent mechanisms. In addition, SNFuc blocks 786 cell passages in the S and G2-M phases of the cell cycle.

## 1. Introduction

Nanoparticles have attracted significant attention due to their various applications in the fields of biotechnology and biomedical sciences. The term nanogel is frequently used to define aqueous dispersions of hydrogel particles composed of nanoscale-sized physically or chemically cross-linked polymer networks [1]. Their application in medicine is very promising since they exhibit high stability and loading capacity, as well as responsiveness to environmental factors such as pH, ionic strength and temperature, making them viable candidates for transport and drug release. However, when nanoparticles enter the bloodstream they are often opsonized by plasma proteins and/or rapidly removed from the blood by the mononuclear phagocytic system [2]. Hydrophobic particles are typically opsonized much faster than their hydrophilic counterparts [3]. Several researchers have focused on the camouflaging or masking of nanoparticle surfaces to avoid these events. As a result, some polymers have been evaluated as protecting groups, including polyacrylamides, polyvinyl alcohol (PVA), poly(ethylene glycol) (PEG) and polysaccharides [4]. 

Recently, self-assembled nanoparticles based on natural polysaccharides have been of particular interest in light of their good biocompatibility, biodegradability, reduced toxic side effects and improved therapeutic effects [5]. Another advantage of using polysaccharides is that these molecules contain reactive groups which can be used to introduce different chemical ligands [6]. For example, polysaccharides can be used to synthesize water-soluble polymers with grafted hydrophobic molecules, that is, amphiphile polymers [7]. Through self-assembly, hydrophobic regions are directed from outside the molecule to form an inner core surrounded by hydrophilic chains. This type of structure is suitable for trapping hydrophobic substances such as hydrophobic drugs [8].

Recently, Bae and colleagues [9] reported that heparin nanogels were able to induce apoptosis in tumor cells. The authors attributed antitumor activity to heparin, a sulfated polysaccharide exhibiting several biological activities. Of these, the most noteworthy and widely studied is anticoagulant activity [10]. Pharmaceutical-grade heparin is derived from porcine intestinal mucosa through several steps of purification. However, the amount of heparin obtained from each animal is small and may be accompanied by impurities, including other sulfated polysaccharides and proteins [11]. Sulfated polysaccharides from seaweed, such as sulfated fucans, are an alternative to heparin. 

Synthesis of nanoparticles from fucans is still new and to the best of our knowledge only one article exists describing this process [12]. Sulfated fucans comprise families of polydisperse polysaccharides based on sulfated L-fucose. Heterofucans are also known as fucoidans and are not widespread in nature, occurring only in brown seaweed and tunicates [13,14], with seaweeds being the most important source of fucans. Seaweed synthesizes fucan and displays unique structural characteristics reflected in the biological, pharmacological and biotechnological properties of the polysaccharide. Furthermore, these structural characteristics can be modified by biotic and abiotic factors that seaweed is exposed to, as well as extraction and purification methods used to obtain sulfated polysaccharides. Recent reviews regarding seaweed fucans have been restricted to their structural characteristics and primary biological/pharmacological activities [15].

**Figure 1:**
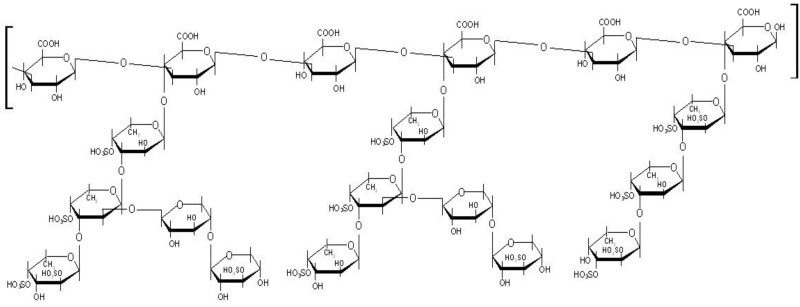
Structure of fucan A from *Spatoglossum schroederi* proposed by Leite and colleagues [16].

The brown seaweed *Spatoglossum schröederi* synthesizes three heterofucans, namely fucan A, fucan B and fucan C. Our group proposes 21 kDa fucan A structure as consisting of a core of β(1–3) glucuronic acid-containing 4.5 kDa oligosaccharide, with branches at C-4 of α(1–3)-linked fucose chains. Fucose is substituted at C-4 and C-2 (minor) with sulfate groups. In addition, some fucose residues are substituted at C-2 with chains of β(1–4) xylose, which, in turn, is also partially sulfated (Figure 1) [16]. This fucan displayed no mutagenicity or genotoxicity [17]. In addition, fucan A shows no toxicity *in vivo* [18]. Thus, in the present study we synthesized and characterized hydrophobically modified fucan A nanogel (SNFuc), assessing their effect on several tumor and normal cell lines. 

## 2. Results and Discussion

### 2.1. FT-IR Analysis

**Figure 2:**
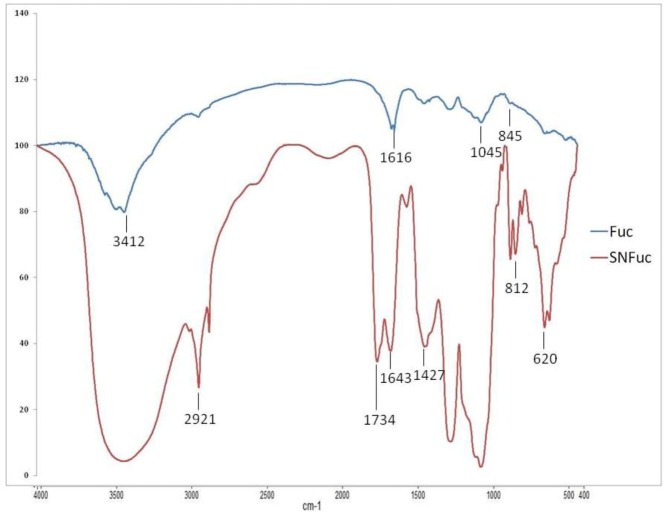
FTIR spectra of native fucan and SNFuc.

The FI-IR analysis of native fucan and SNFuc is showed in Figure 2. Characteristic sulfate absorptions were identified in the FT-IR spectra of compounds: bands around 1274 cm^−1^ for asymmetric S=O stretching vibration and bands around 1045 cm^−1^ for symmetric C–O vibration associated with a C–O–SO_3_ group. The peaks at 810–850 were caused by the bending vibration of C–O–S [19]. At 3000–3400 cm^−1^ Fuc A and SNFuc showed bands from the stretching vibration of O–H and C–H, respectively [20]. However, the SNFuc FI-IR spectrum showed the intensity of these bands increased due the presence of N–H (3000–3400 cm^−1^) and stretching vibrations of CH_2_ in hexadecyl residues (2921 and around 2850 cm^−1^) [21]. The peak of the C–H symmetric deformation vibration was at 1427 cm^−1^ [22]. The intensities of this absorption band increased with chain length of the CH_2_ groups in SNFuc. A band at 1616 cm^−1^ was identified only in fucan A spectrum and was assigned to antisymmetric stretching vibration of COO^−^ of glucuronic acid [23]. The presence glucuronic acid was also confirmed with a symmetric vibration peak around 1410 cm^−1^. On the other hand, SNFuc spectrum showed a band at 1740 cm^−1^ caused by C=O stretch vibrations in COOH and esters [24]. The band at1643 cm^−1^ was due the amine I vibration which is overlapped with the vibration of water. Less intense peak around1550 cm^−1^ arose from amide II vibration in alkylamides and thus confirmed amidation. Additionally, band at 620 cm^−1^ was assigned to N–C=O bending vibration [24]. 

### 2.2. ^1^H NMR and Elemental Analysis

**Figure 3:**
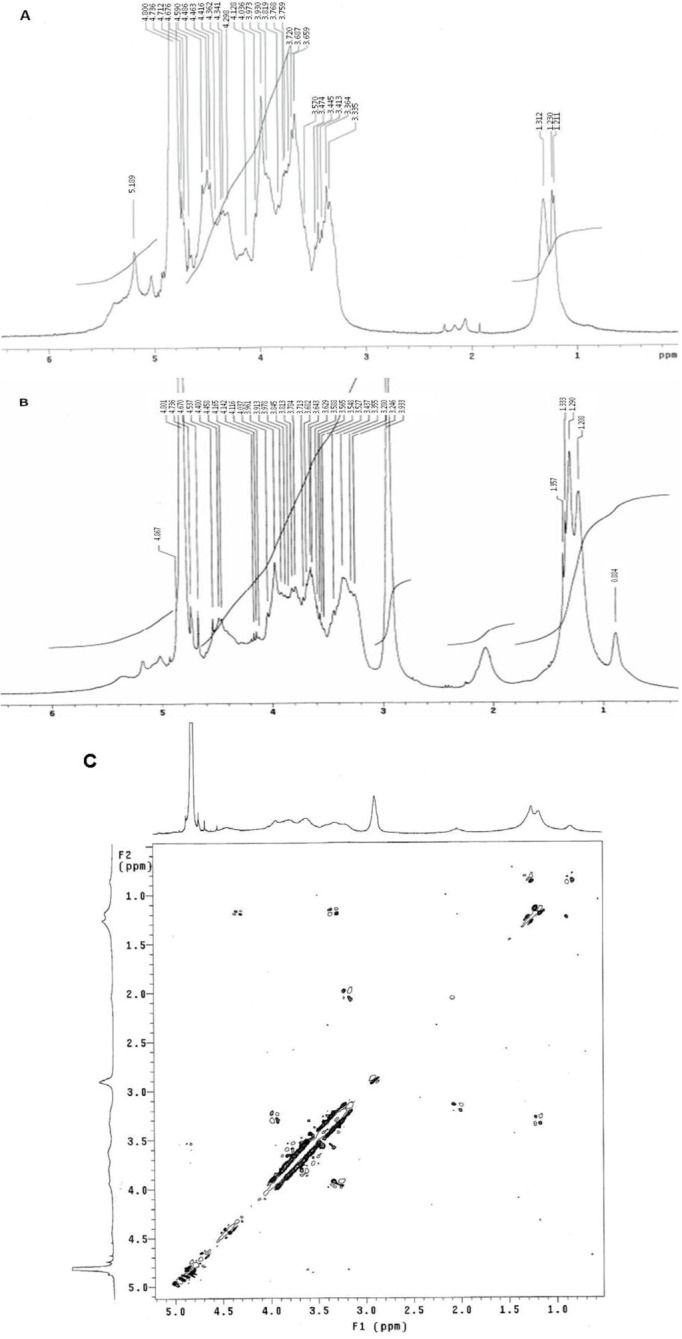
^1^H NMR spectra of native fucan (**A**) and SNFuc in D_2_O at 25 °C (**B**) and Correlation Spectroscoy analysis (COSY spectrum) of SNFuc (**C**).

The native fucan A ^1^H NMR spectrum is shown in Figure 3A. The two main α-anomeric protons, which correspond to α-L-fucose units, were observed at 5.18 and 5.08 ppm. In contrast with the simplicity of both α-fucose residues, β systems show a certain degree of multiplicity, likely due to diversity in the positions of interglycosidic linkages of sugar residues. β-Anomeric protons appeared as two unresolved multiplets centered at 4.4 and 4.9 ppm, together with signals of protons from sulfation sites, whereas the remaining fucan A protons appear in the range of 3.5–4.5 ppm [16]. Signals around 1.2–1.4 ppm were assigned to CH_3_ protons of fucose residues.

Figure 2B depicts the ^1^H NMR of SNFuc. The reaction between fucan and hexadecylamine follows a Michael addition mechanism. Signals between 5.6 and 1.0 ppm in the ^1^H NMR spectrum of SNFuc are assigned to protons from the fucan A scaffold. Proton peaks from hexadecylamine are observed at 2 ppm and at 1.0–1.3 ppm. These overlap the signals of CH_3_ groups from fucose residues (Figure 3B) [25]. The proton signal near 3 ppm is attributive to neither the fucan nor the hexadeyilamine and thus it seems to arise from a contaminant that could not be eliminated by extensive 2 dialyses. According to COSY experiment (Figure 3C), this signal does not correlated with those of polysaccharide or substituent.

Due to the overlap of the peaks arising from the fucan and the hexadecylamine protons, it was not possible to determine the degree of substitution (DS) using the ^1^H NMR spectrum analysis. Nonetheless the ^1^H NMR spectrum confirms the chemical grafting of hexadecylamine molecule to the fucan structure, as evidenced by the hexadecylamine peaks.

Therefore the degree of substitution (DS) was estimated through the SNFuc elemental analysis, based on the ratio of amide nitrogen and the sulfate sulfur from the fucans. This resulted in a N/S ratio of 0.66 (2.9/4.4). Taking into account the proposed structure of the fucan and the corresponding theoretical elemental composition, a degree of substitution superior to 100% is obtained. Indeed, according to the structure shown in Figure 1, fucan has 14 sulfate groups per 6 carboxylic groups. Thus, if 100% of the carboxylic groups react with an hexadecylamine, we would end up with a ratio of 6N/14S, therefore our experimental N/S ratio corresponds to a degree of substitution above the maximum theoretical value. This may be interpreted as significant of a very high degree of substitution, probably close to 100%, the value obtained being certainly overestimated due to heterogeneity of the fucan; the theoretical structure used in this estimation may not accurately correspond to the material used.

### 2.3. Formation of Nanoparticles

Dissolution of SNFuc in water is expected to give rise to micelle formation, owing to the amphiphilic nature of the molecule. Nanogel formation was assessed by Dynamic Light Scattering (DLS).

**Figure 4:**
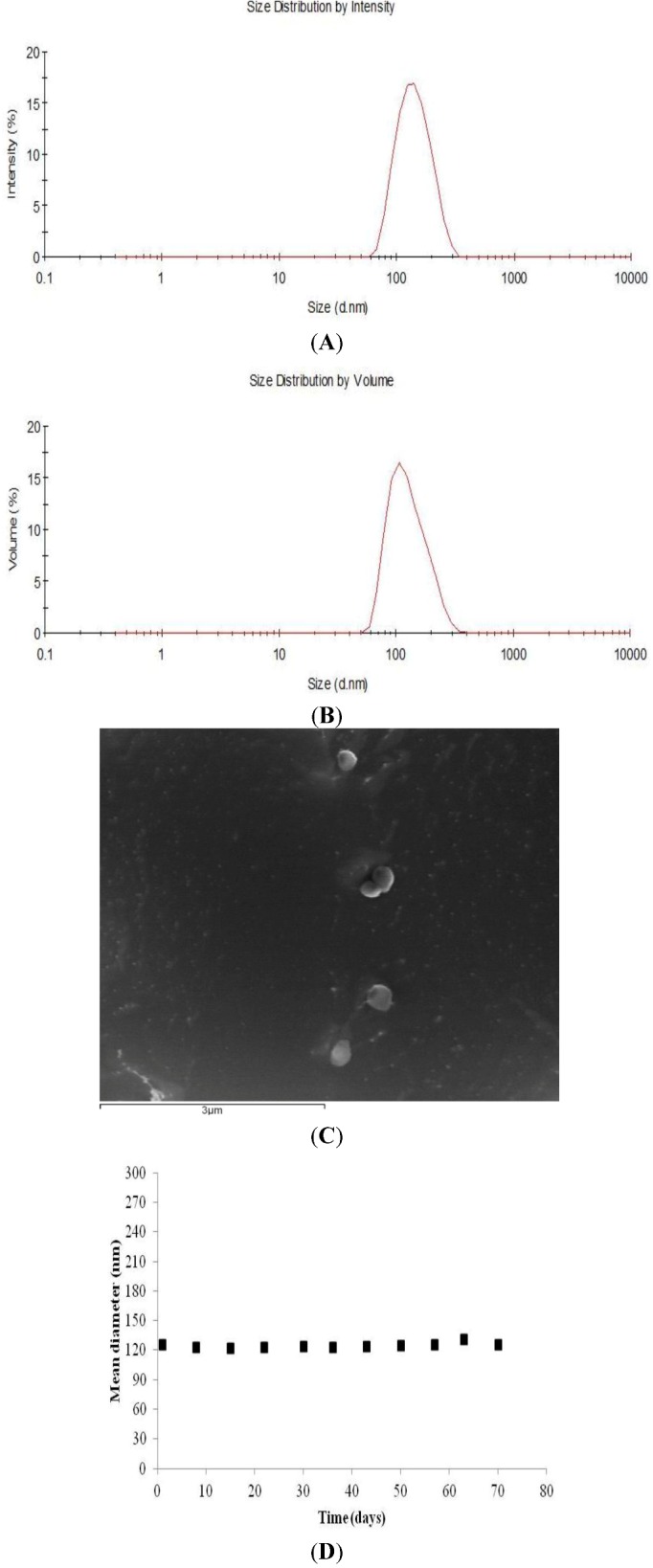
Particle size distribution (**A**) and volume (%) (**B**) of SNFuc nanogels (0.1 g/dL) measured by dynamic light scattering (DLS) and Fucan nanogels observed by Cryo-SEM (**C**). The figures represent data obtained from the determination of fucan nanoparticle properties prepared in three different experiments; (**D**) Colloidal stability of nanoparticles in water. The SNFuc solution (0.1 g/dL) was stored at 25 °C for up to 70 days. The error bar represents standard deviation (*n* = 5).

DLS analysis provides valuable information on the homogeneity of dispersion. SNFuc (0.1 g/dL) in aqueous media had a mean diameter of 123 nm, with unimodal size distribution and a corresponding average polydispersity index of 0.269 (Figure 4A). Since size distribution intensity is somewhat influenced by the presence of larger particles, whereas volume distribution better characterizes the more representative population, we also analyzed the volume distribution of nanoparticles. SNFuc volume analysis also demonstrated the homogeneity of nanoparticles (Figure 4B). The observation of the nanogel by CryoSEM provides images of particles in the same size range as detected by DLS (Figure 4D).

### 2.4. Nanogel Stability

Present and future nanogel applications require a high degree of control over properties, among which, particle stability in aqueous solutions is one of the most sought after since this would facilitate its biomedical application. Analysis of nanoparticle stability in a time interval allows us to determine whether molecule characteristics are ideal for biomedical applications [26,27].

The magnitude of zeta potential gives an indication of colloidal system stability. Particles in a suspension with a large zeta potential value, whether negative or positive, will repel each other and as such do not aggregate. However, if the particles exhibit low zeta potential value (close to zero), there is no electrostatic force to prevent particles from aggregating. Nanogel has a zeta potential of −38.3 ± 0.74 mV and is therefore expected to be stable in aqueous suspension [28].

Assessing changes in nanoparticle size over a period of time is a good indicator of its stability. Thus, in order to evaluate the stability of SNFuc nanoparticles, we established their size by DLS, at 25 °C, for up to 70 days (Figure 4C). Data showed indicated high colloidal stability of nanoparticles in an aqueous medium.

### 2.5. Effect of SNFuc on Cell Proliferation

**Figure 5:**
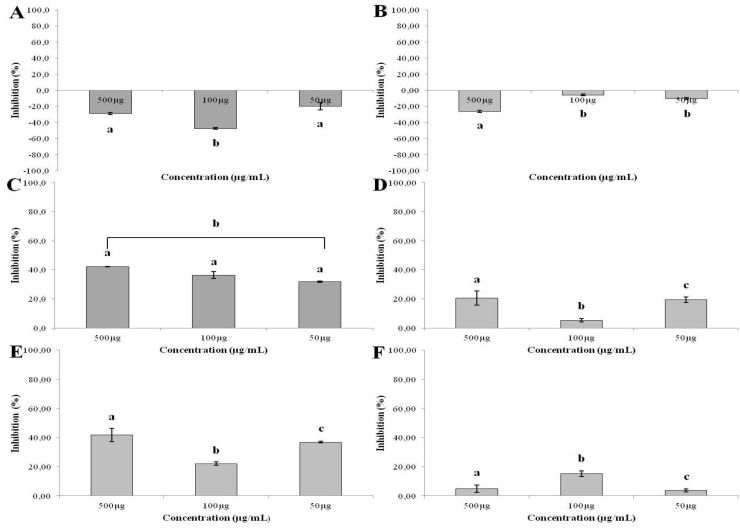
Effect of SNFuc nanogel on Chinese hamster ovary (CHO) (**A**); mouse monocyte macrophage (RAW) (**B**); human renal cell carcinoma (786) (**C**); human hepatocellular carcinoma (HepG2) (**D**); rabbit aorta endothelial cells (RAEC) (**E**) and human marrow stromal cell line (HS-5) (**F**) cell proliferation, after 24 h of incubation in different concentrations (50, 100 and 500 µg/mL). Results are expressed as percentage of control cells normalized to 100% in the absence of polysaccharide (mean ± SD of seven determinations). Different letters indicate a significant difference between concentrations of individual sulfated polysaccharides (*p* < 0.05).

**Figure 6:**
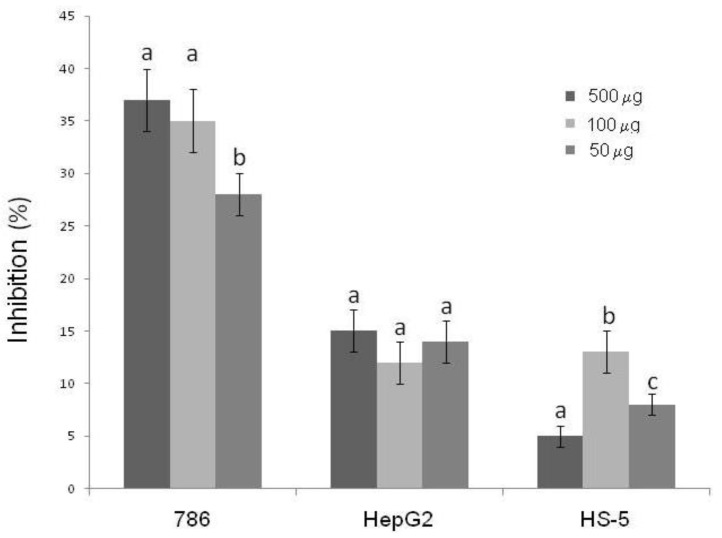
Effect of SNFuc on tumor cells proliferation. The rate of cell proliferation inhibition was determined using BrdU. Results are expressed as percentage of control cells normalized to 100% in the absence of polysaccharide (mean ± SD of four determinations). Different letters indicate a significant difference between concentrations of individual sulfated polysaccharides (*p* < 0.05).

Antiproliferative activity of SNFuc on CHO, RAW, 786, HepG2, RAEC and HS-5 cells was investigated using the colorimetric MTT assay. Proliferation of non-tumor cells CHO and RAW (Figure 4A,B) was stimulated by the presence of SNFuc, after an incubation period of 24 h. On the other hand, SNFuc showed antiproliferative activity when RAEC cells and tumor cells were used (Figure 5). Additionally, our data suggest that cytotoxicity of fucan nanogel was cell-specific, arising from cell-fucan nanogel interaction. In Figure 6 the SNFuc antiproliferative activity against 786, HepG2 and HS-5 cells was further confirmed using BrdU incorporation as described in the Experimental Section. Proliferation of the 786 tumor cell was the most affected one, showing an inhibition rate of approximately 40% in the presence of SNFuc. This cell line was therefore used in additional tests.

### 2.6. Cell and Nuclear Morphology Change

**Figure 7:**
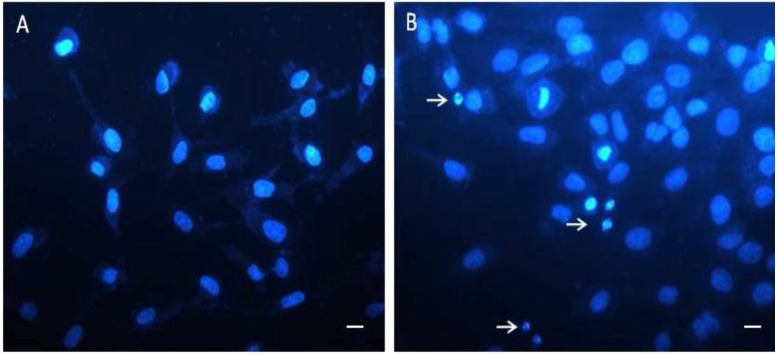
Micrograph of 786 cells treated with SNFuc. These cells were incubated with 0.5 mg/mL SNFuc for 24 h and labeled with DAPI to show nuclear morphology. (**A**) Control 786 cells, without SNFuc; (**B**) 786 cells treated with SNFuc, showing nuclear morphological changes such as pyknosis (arrows). Bar, 10 µm.

The 786 cells were exposed to SNFuc (0.5 mg/mL) for 24 h. Contrast microscopy showed that most cells became circular (Data not shown). Nuclear morphological changes were observed by DAPI staining. In the control group, 786 cells were rounded and homogeneously stained (Figure 7A). After 24 h of treatment with SNFuc, blebbing nuclei, pycnotic bodies, morphological alterations and granular apoptotic bodies appeared (Figure 7B). Marked apoptotic morphologic alterations, including nuclear condensation, suggest nanoparticles induce apoptosis in 786 cells.

### 2.7. Balance between Apoptosis and Necrosis

**Figure 8:**
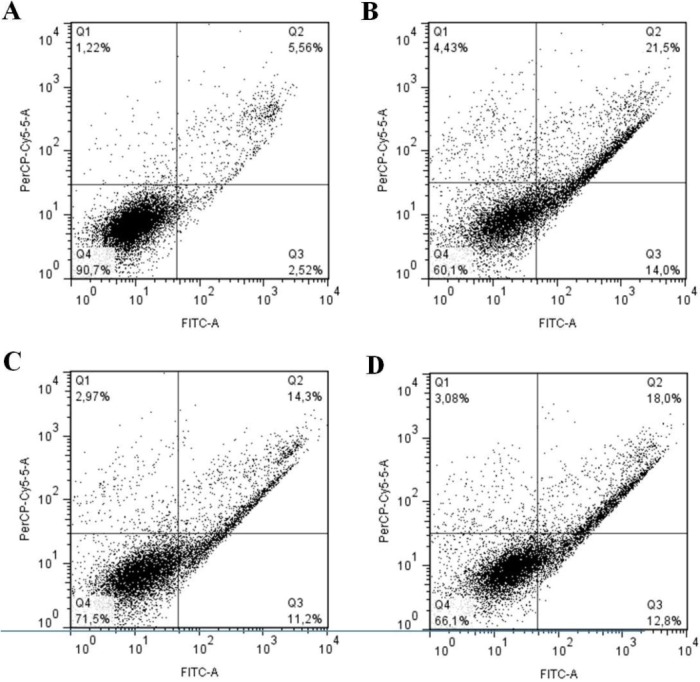
Changes in the percentage of normal, apoptotic and necrotic cells after SNFuc-TBA nanogel incubation. (**A**) Control; (**B**) Cells maintainedin the presence of0.5mg/mLSNFuc-TBA; (**C**) Cells kept in the presence of0.5mg/mLSNFuc and ZVAD-FMK; (**D**) Cells maintainedin the presence of0.5mg/mLSNFuc and E-64. After 24 h, cells were treated withAnnexinV/PI and analyzed by Flow Cytometry. Q1: AnnexinVnegative/PIpositive;Q2:AnnexinV/PIpositive;Q3:AnnexinVpositive/PI negative;Q4:AnnexinV/PInegative. Analysis flow cytometry data was performed using FlowJosoftwarev.7.6.3. Similar results were obtained in three independent experiments.

In order to observe additional influences of SNFuc nanogel on 786 cell lines, apoptotic and necrotic status were analyzed. SNFuc (0.5 mg/mL) significantly (*p* < 0.001) increased the percentage of cells in early (from 2.5 to 14.0%) and late apoptosis (from 5.5 to 21.5%), whereas the percentage of necrotic cells was not significantly affected (Figure 8B). 

The most widely studied fucan with regard to its pro-apoptotic mechanism of action is the *Fucus vesiculosus* fucan, known as fucoidan. This fucan promotes caspase activation, inducing apoptosis of human lymphoma HS-sultan cells. However, when these cells were concomitantly incubated with fucoidan and a caspase inhibitor, the fucoidan effect was partially offset [29]. Thus, in order to determine the role of caspases in fucan A nanogel induced apoptosis, 786 cells were pre-incubated with Z-VAD-FMK (pan-caspase inhibitor). This was followed by stimulation with 0.5 mg/mL SNFuc nanogel for 24 h and FACS analysis. Z-VAD-FMK modestly inhibited the fucan nanogel-induced late apoptosis to 14.3% (Figure 8C). Z-VAD-FMK did not completely inhibit the SNFuc nanogel effect. A synthetic inhibitor of cysteine proteinase (E-64) was also used, but did not alter the fucan nanogel effect on 786 cells (Figure 8D).

**Figure 9:**
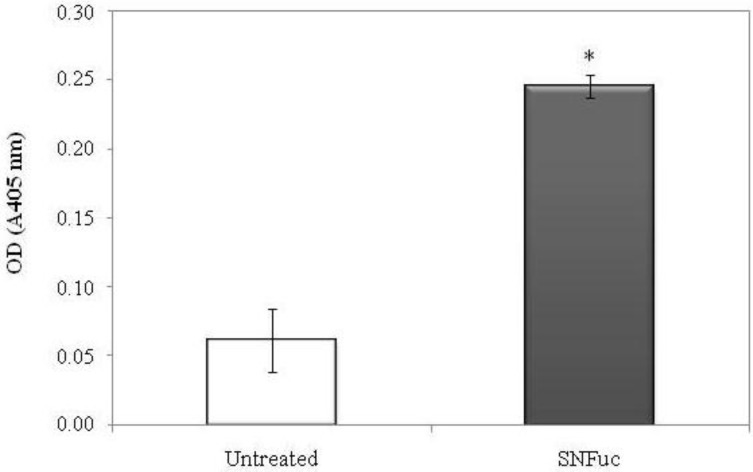
Effect of SNFuc (0.5 mg/mL) on caspase-3 activity in 786-0 cells after 24 h of treatment. *p* < 0.001 *vs*. negative control (untreated cells).

In addition caspase-3 activation was examined in 786 cells, which were treated with or without SNFuc. Data in Figure 9 indicate that SNFuc promoted activation of caspase-3 in 786 cells. Several authors have shown fucan induces cell apoptosis by activated caspase-3 [29,30], as observed with SNFuc. However, the data from Figure 8 and Figure 9 support the proposition that fucan nanogel induces apoptosis through both caspase and caspase-independent mechanisms. 

Research conducted on fucans from brown seaweed give an indication of how pro-apoptotic fucans behave. In most cases, apoptosis induction is related to caspase activation. However, other proteins involved in cell survival pathways may be affected by the presence of SP in the culture medium. For example, the heterofucan from *Undaria pinnatifida* induced apoptosis in A549 human lung carcinoma cells through down-regulation of anti-apoptotic protein Bcl2 and activation of the caspase pathway. This heterofucan also down-regulated p38MAPK and PI3K/Akt and activated the ERK1/2MAPK pathway [31].

There are reports that caspase activation may not be necessary for apoptosis induction by some SPs. A recently published study showed a heterofucan with pro-apoptotic activity extracted from the seaweed *Sargassum filipendula*, denominated SF-1.5V. This SP inhibited HeLa (adenocarcinoma cervical cell) cell proliferation by apoptosis induction through a caspase-independent mechanism. SF-1.5v induces apoptosis in HeLa mainly by mitochondrial release of an apoptosis-inducing factor (AIF) into cytosol. In addition, SF-1.5v decreases the expression of Bcl-2 and increases expression of the apoptogenic protein Bax [14].

There are no data available regarding the antiproliferative mechanism of fucan nanogel. Further studies are therefore needed to clarify their mechanism, particularly with respect to SNFuc, which exhibited the strongest antiproliferative activity.

### 2.8. Cell Cycle Analysis

The antiproliferative activity of fucans may also be a result of their interference in the cell cycle. Fucans from the seaweeds *Turbinaria ornate* [32] and *Bifurcaria bifurcata* inhibit proliferation of lung carcinoma (NSCLC-N6) by blocking its passage in the G1 phase of the cell cycle [33]. This effect was also observed with sulfated fucans from the seaweed *Ascophyllum nodosum*, which inhibit proliferation of NSCLC-N6 cell lines by blocking cell growth in the G1 phase [34]. As such, we also analyzed the effect of fucan nanogel on the 786 cell cycle.

**Figure 10:**
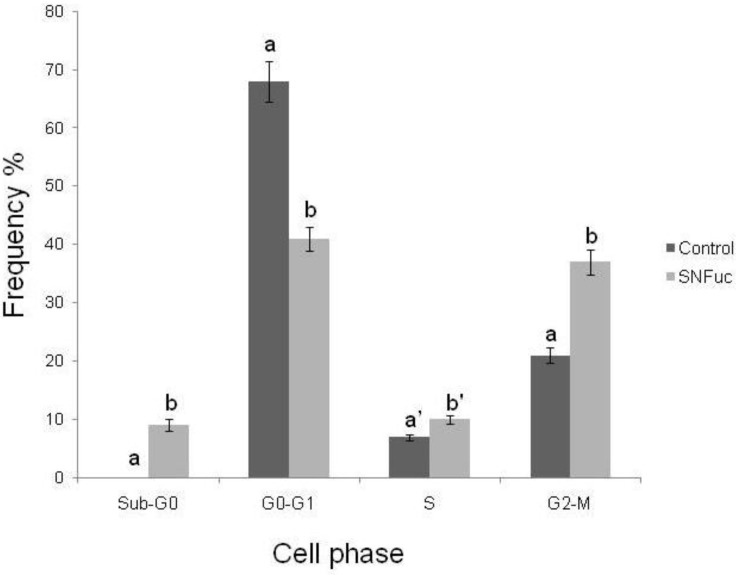
Evaluation of cell-cycle arrest after exposure to SNFuc. After 24 h of treatment with SNFuc (0.5 mg/mL), 786 cells were fixed, treated with RNase, stained with PI and analyzed by flow cytometry to assess cell cycle distribution. Control cells without SNFuc. Cell cycle arrest data were analyzed using FlowJo software v. 7.6.3. Similar results were obtained in three independent experiments. Different letters indicate a significant difference between concentrations of individual sulfated polysaccharides (a and b, *p* < 0.001; a′ and b′, *p* < 0.05).

Analysis of the cell cycle after treatment of 786 cells with the SNFuc showed that, during incubation in the nanogel, the proportion of G0/G1 phase cells was reduced (Figure 10), and the percentage of cells at the S and G2/M phases of the cell cycle grew. This was accompanied by the appearance of a large number of apoptotic cells in the sub-G0/G1 phase. Data indicate that cells in the G1 phase during incubation entered the subsequent phase and stopped at this point. If cells entered apoptosis from the G1 phase, there was no accumulation of cells in S and G2/M phases. 

## 3. Experimental Section

### 3.1. Materials

Tetrabutylammonium fluoride trihydrate (TBA) and dimethyl sulfoxide (DMSO, H_2_O ≤ 0.005%), were obtained from Fluka (Buchs, Switzerland) and deuterium oxide (D_2_O) from Aldrich. Regenerated cellulose tubular membranes, with 12,000 MWCO, were obtained from Membrane Filtration Products and hexadecylamine, 1-ethyl-3(3-dimethylaminopropyl) carbodiimide (EDC) and *N*-hydroxysuccinimide (NHS) were obtained from Sigma-Aldrich Co. (St. Louis, MO, USA). Water used for synthesis and characterization was purified by distillation, deionization and reverse osmosis (Milli-Q Plus). All other chemicals and solvents were analytical grade and used as received.

Fucan A from *Spatoglossum schroederi* was obtained as described by Almeida-Lima and colleagues [18] and its purity was determined by ^1^H RMN.

### 3.2. Synthesis and Characterization of Fucan Conjugates

To obtain nanogels from fucan using the methodology applied by Oudshoorn *et al.* [25], we initially dissolved the fucan using Tetrabutylammonium Fluoride Trihydrate (TBA) in order to activate the fucan carboxyl groups. Moreover, TBA was also chosen since any excess can be easily eliminated from the reaction medium by dialysis against a NaCl solution. This step is important because fucans are not soluble in DMSO, the solvent used to produce nanoparticles. 

To render fucans soluble in DMSO, the fucan linked sodium ions were exchanged with the lipophilic tetrabutylammonium (TBA) ion using ion exchange resin (Dowex^®^ 50W-X8 cation exchange resin). The Fucan-TBA solution was lyophilized and used for chemical modification with hexadecylamine.

**Figure 11:**
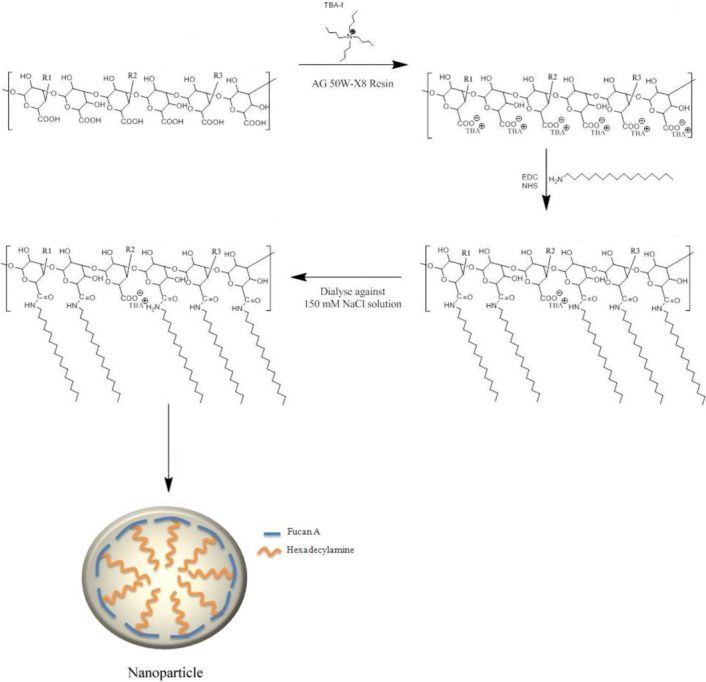
Synthetic scheme of fucan A nanogel synthesis. TBA: Tetrabutylammonium Fluoride Trihydrate; EDC: 1-ethyl-3(3-dimethylaminopropyl) carbodiimide; NHS: *N*-hydroxysuccinimide; R1, R2 and R3: side chain of fucan A (see Figure 1)*.*

Hydrophobic hexadecylamine was chemically conjugated to the backbone of Fucan-TBA in the presence of EDC and NHS. Briefly, Fucan-TBA was dissolved in DMSO and the reaction mixture was stirred for one day. Next, EDC, NHS and hexadecylamine were added and the reaction mixture was stirred for one day. The resulting solution was first dialyzed in a 150 mM sodium chloride solution for 2 days, and then in dH_2_O for 3 days, in order to remove TBA and substitute it for the original Na^+^ ion. SNFuc derivative was obtained as a white powder (Figure 11). ^1^H NMR two-dimensional Correlation Spectroscoy analysis and elemental analysis was performed to analyze the structure of the reaction product.

### 3.3. SNFuc Degree of Substitution

The degree of substitution (DS) was estimated by the evaluation of N/S ratio as determined by elemental analysis, taking in account the fucan structure proposed in Figure 1. Considering the theoretical proportion of carboxylic to sulfate groups, DS is calculated based on the N/S ratio (proportional to the amide/sulfate ratio).

### 3.4. Preparation of Self-Assembled Nanoparticles

Lyophilized SNFuc was dissolved in ultra-pure water under stirring, at 50 °C, until a clear solution was obtained. In the range of SNFuc used to prepare a 1 g/dL solution, 3 h of stirring is the maximum time required to dissolve SNFuc. The solution of self-assembled nanoparticles was then filtered through a 0.45 µm filter and stored at room temperature.

### 3.5. Size Distribution, Zeta Potential and CryoSEM Observation

Size distribution, zeta potential and nanoparticle volume were determined with a Malvern Zetasizer, NANO ZS (Malvern Instruments Limited, UK), using an He-Ne laser (wavelength of 633 nm) and detector angle of 173°. Size distribution and zeta potential were established through dynamic light scattering (DLS).

For size distribution measurements, a dispersion of nanoparticles in ultra-pure water (1 mL) was analyzed at 25 °C in a polystyrene cell. Nanoparticle concentration was adjusted by dilution with ultrapure water with concentrated nanoparticle dispersion.

The fucan nanogel concentrated by ultrafiltration (Amicon Ultra-4 Centrifugal Filter Units, cutoff molecular weight 1 × 10^5^) was negatively stained with phosphotungstic acid (Riedel-de Haën; 0.01% w/v) and observed by Cryo-scanning electron microscopy (cryoSEM), using an electronic microscope (SEM/EDS: FESEM JEOL JSM6301F/Oxford Inca Energy 350).

DLS cumulant analysis characterizes samples using the mean value (z-average) for size and a width parameter known as polydispersity, or the polydispersity index (PdI). The z-average diameter is the mean hydrodynamic diameter, determined by the intensity of scattered light. Fundamental size distribution generated by DLS is an intensity distribution, which can be converted to volume distribution using Mie theory. Volume distribution can in turn be further converted to a number distribution. The present study considers the z-average as the best approach for actual nanoparticles size. Each sample was analyzed in a folded capillary cell. Zeta potential values were calculated using the Smoluchowski equation. Repeated measurements were performed (3 times) and values reported are average values.

### 3.6. ^1^H NMR and Elemental Analysis

Lyophilized reaction products were dispersed in D_2_O (10 mg/mL), as were nanoparticles and fucan (10 mg/mL). Samples were stirred overnight at 50 °C to obtain a clear dispersion, which was transferred to 5 mm NMR tubes. One-dimensional ^1^H NMR and two-dimensional correlation spectroscopy (COSY) measurements were performed with a Varian Unity Plus 300 spectrometer operating at 299.94 MHz. 

Elemental analysis was performed using a Leco CHNS 932. The sample was lyophilized and analyzed in the solid state.

### 3.7. Antiproliferative Activity

The present study used monocyte macrophage (RAW 264) cells, Chinese hamster ovary (CHO) cells, renal cell carcinoma (786-0) cells, hepatocellular carcinoma (HepG2) cells, and aorta endothelial cells (RAEC). Cells were grown in F-12 (RAEC) or DMEM (all others) medium supplemented with 10% newborn calf serum (Cutilab, Campinas-SP, Brazil) and penicillin-streptomycin (1 mg/mL; Sigma-Aldrich, St Louis, MO, USA), and incubated at 37 °C in 5% CO_2_. Cell viability was assessed using trypan blue (Sigma).

Cell cultures were grown in 75 cm^2^ flasks in DMEM medium. Cells were seeded into 96-well plates at a density of 5 × 10^3^ cells/well and allowed to attach overnight in 300 µL medium incubated at 37 °C, 5% CO_2_. The fucan nanogel was added at a final concentration of 0.05/0.1 and 0.5 mg/mL for 24 h, at 37 °C and 5% CO_2_. After incubation, traces of nanogel were removed by washing the cells twice with 200 µL PBS and applying 100 µL of fresh medium and 10 µL of 12 mM MTT dissolved in PBS to determine the effects of the fucan nanogel on cell proliferation. Cells were then incubated for 4 h at 37 °C and 5% CO_2_. To solubilize the product of MTT cleavage, 100 µL of isopropanol containing 0.04 N HCl was added to each well and thoroughly mixed using a multichannel pipette. Within 1 h of HCl-isopropanol addition, absorbance at 570 nm was read using a Multiskan Ascent Microplate Reader (Thermo Labsystems, Franklin, MA, USA). The percentage of cell proliferation inhibition was calculated as follows:


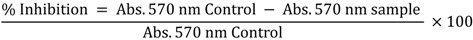


In addition the tumor cells proliferation in the presence of SNFuc was also determined after 4 h of BrdU incorporation. Cells were seeded into 96-well plates at a density of 5 × 10^3^ cells/well and allowed to attach overnight in 300 µL medium incubated at 37 °C, 5% CO_2_. The fucan nanogel was added at a final concentration of 0.5 mg/mL for 24 h, at 37 °C and 5% CO_2_. After incubation, traces of nanogel were removed by washing the cells twice with 200 µL PBS and the BrdU incorporation was determined accordance with the manufacturer’s instruction (BrdU cell proliferation assay kit, Cell Signaling, Danvers, MA, USA).

### 3.8. Fourier Transformed Infrared Spectroscopy (FT-IR)

The compound (Fucan A and SNFuc) (20 mg) were mixed thoroughly with dry potassium bromide. A pellet was prepared and the infrared spectrum was measured on a Thermo Nicolet spectrometer instrument, model Nexus 470 FT-IR, between 500 and 4000 cm^−1^. Thirty-two scans at a resolution of 4 cm^−1^ were averaged and referenced against air [35]. 

### 3.9. Annexin V-FITC/PI Double Staining and Analysis by Flow Citometry

In order to evaluate the effects of SNFuc on cell death, a FITC/Annexin V Apoptosis Kit was used, with Dead Cell Annexin FITC and PI, for Flow Cytometry (Invitrogen, Catalog no. V13242). Cells were grown in 6-well plates until they reached confluence of 2 × 10^5^ cells/mL and were then stimulated to enter G0 in a medium without serum for 48 h. Next, cells were to exit G0 by adding DMEM supplemented with 10% FBS, in the presence of SNFuc (0.5 mg/mL) or not. A negative control was prepared without the presence of SNFuc, and the action of SNFuc incubated with pan-caspase inhibitor Z-VAD-FMK (carbobenzóxy valyl-alanyl-aspartyl-[*O*-methyl]-fluorometilcetone) was also tested (0.02 mM). After 24 h, 786 cells were trypsinized, collected and washed with cold PBS. The supernatant was discarded and cells were resuspended in 200 µL of 1× Binding Buffer. Five microlitres of Annexin V-FITC and 1 µL of PI solution 100 µg/mL was added in 100 µL of cell suspension. Cells were incubated for 15 min at room temperature and kept under light protection. After the incubation period, 400 µL of binding buffer for annexin V 1× was added and cells were analyzed by flow cytometry (flow cytometer FASCANTO II, BD Biosciences), measuring fluorescence emission at 530–575 nm for annexin V and 630/22 nm for PI. FlowJo software v. 7.6.3 (Tree Star, Inc., CA, USA) was used for data analysis as described in Rabelo and colleagues [36].

### 3.10. Cell Cycle Analysis

786 cells were washed with cold PBS and the supernatant was discarded. The pellet of cells was then incubated with 2% paraformaldehyde, washed with cold PBS and permeabilized with 0.01% saponin for 15 min. After this procedure, cells were incubated with 10 µL of RNase (4 mg/mL) at 37 °C for 30 min. Five microlitres of PI solution (25 mg/mL) and 200 µL of cold PBS were added and cells were transferred to a flow cytometer for analysis of cell cycle arrest (630/22 nm). The percentage of apoptotic cells was determined every 20,000 events and graphs obtained in the experiment represent data from three independent experiments. FlowJo software v. 7.6.3 (Tree Star, Inc., CA, USA) was used for data analysis [36].

### 3.11. Caspase-3 Activity Assay

786-0 cells were placed in a petri dishes (1 × 10^6^ cells/mL), and after 24 h of incubation, cells were incubated with SNFuc and washed after 24 h in ice-cold PBS and scraped into 200 mL lysis buffer [50 mM Tris-HCl (pH 7.4), 1% Tween 20, 0.25% sodium deoxycholate, 150 mM NaCl, 1 mM EDTA, 1 mM Na_3_VO_4_, 1 mM NaF], and protease inhibitors [1 mg/mL aprotinin, 10 mg/mL leupeptin and 1 mM 4-(2-aminoethyl)benzenesulfonyl fluoride] for 2 h 4 °C. The same conditions were used for untreated cells. Protein extracts were cleared by centrifugation and protein concentrations were determined using Bradford reagent with bovine serum albumin as standard. *In vitro* caspase-3 protease activity was measured using a caspase activation kit according to the manufacturer’s protocol (Invitrogen, Carlsbad, CA, USA). 50 µL of cell lysate (200 µg of protein) was mixed with 50 µL of 2× reaction buffer [containing 10 µL of 1 M dithiothreitol and 5 µL of 4 mM synthetic tetrapeptide Asp-Glue-Val-Asp conjugated top-nitroanilide (pNA)] in 96-well plate, after which the mixture was incubated for 2 h at 37 °C in the dark. Active caspase cleaves the peptide and releases the chromophore pNA that can be detected at 405 nm. Theoretically, the apoptotic cell lysates containing active tested caspases should yield a considerable emission compared with the non-apoptotic cell lysates. Data presented are representative of those obtained in at least three independent experiments done in duplicates.

### 3.12. Statistical Analysis

Mean and standard-error of the mean (SEM) were calculated for all quantitative data. For antiproliferative assays, data were analyzed by one-way analysis of variance (ANOVA). When signiﬁcant intergroup differences occurred, these were assessed by Student-Newmans-Keuls post-tests for multiple group comparison. In all cases, statistical signiﬁcance was set at *p* < 0.05 or <0.001.

## 4. Conclusions

The present study prepared and characterized a nanogel, formed by an inner hydrophobic core (represented by hexadecylamine), surrounded by hydrophilic chains (represented by sulfated fucan). The resulting fucan nanogel exhibited a robust structure in aqueous solution, which was stable for several weeks. Fucan nanogel inhibits 786, RAEC, H-S5 and HepG2 cell proliferation. In addition, this nanogel induces 786 cell deaths by inducing apoptosis through dependent and independent mechanisms of caspases activation and by blocking 786 cell passages in the S and G2/M phases of the cell cycle. Since the fucan nanogel demonstrated an antiproliferative effect against tumor cells, its use, in conjunction with anti-tumor drugs either transported by nanogel or not, shows significant potential in the treatment of different cancers.
